# Improvements of the Surgical Technique on the Established Mouse Model of Orthotopic Single Lung Transplantation

**DOI:** 10.1371/journal.pone.0081000

**Published:** 2013-11-21

**Authors:** Zhikun Zheng, Jianjun Wang, Xia Huang, Ke Jiang, Jun Nie, Xinwei Qiao, Jinsong Li

**Affiliations:** 1 Department of Thoracic Surgery, Union Hospital, Tongji Medical College, Huazhong University of Science and Technology, Wuhan, Hubei Province, China; 2 Institute of Organ Transplantation, Tongji Medical College, Huazhong University of Science and Technology, Key Laboratory of Organ Transplantation, Ministry of Education, Wuhan, Hubei Province, China; University of California Los Angeles, United States of America

## Abstract

**Background:**

A wide range of knockout and transgenic murine models for the study of nonimmune and immune mechanisms in lung transplants are available nowadays, but the microsurgical techniques are difficult to learn. We describe methods to simplify techniques and facilitate learning.

**Methods:**

Traditional procedures were implemented to perform lung transplants in 30 cases (group 1). Improved techniques which included cuff without tail, broadening of the cuff diameter for bronchus, establishment of one tunnel between three structures, innovative technology of the vascular anastomosis and placement of the chest tube post-operation were used to perform lung transplants in 30 cases (group 2).

**Results:**

The improved techniques considerably shorten operative times (96.75±6.16 min and 85.32±6.98 min in groups 1 and 2, respectively). The survival rates in the recipient animals were 86.7% and 96.7% in groups 1 and 2, respectively. Chest X-rays and macroscopic changes of transplanted recipients showed that grafts were well inflated on postoperative day 30. There was no significant difference of the arterial oxygen tension (PaO_2_) between two groups (115.9±7.11 mm Hg and 116.3±6.87 mm Hg in groups 1 and 2, respectively). Histologically, no lung injury was seen in grafts.

**Conclusions:**

We described the modified procedures of orthotopic left lung transplants in mice, which could shorten operative time and increase survival rate.

## Introduction

It has been accepted that Lung transplant (LT) can be regarded as an important therapy for patients with end-stage pulmonary disease. Advances in clinical LT rely on observations made in animal models and many experimental murine LT models have been used. Since the first orthotopic LT in the rat model was reported in 1971 [1], much effort has been done to developing this model [2,3]. In 2007, a mouse LT model was reported by Okazaki [4] which provided the opportunity to study a wide range of knockout and transgenic models for the first time. In 2009, Jungraithmayr [5] described the operative steps involved in procedure.

We have been using the cuff technique for several years in the rat LT model [6-8]. Based on our experience with orthotopic LT in rats, we recently have successfully established the mouse orthotopic LT model [9]. In this study, we report on the details of improvements we have made in surgical techniques that facilitate the orthotopic LT and decrease complications. We also introduce modifications in anastomosis which apparently reduce the operative time. These improvements are highly reproducible in the mouse LT.

## Materials and Methods

### 2.1: Animals

Specific pathogen-free male inbred C57BL/6 mice were purchased from Wuhan University Center for Animal Experiment. Eight- to ten-week-old animals weighing 25±3 g were used both as donors and recipients. Sixty LTs were performed for two groups: the initial 30 LTs in the animals designated as group 1—the procedures reported previously were used. In the subsequent 30 LTs, we used improved techniques.

### 2.2: Ethics Statement

This study was approved by the Experimental Animal Ethics Committee of Tongji Medical College, Huanzhong University of Science and Technology (No. 2011-S252). All animals of this study were treated in accordance with the Principles of Laboratory Animal Care. All samples collected after LTs in this study were used for other animal experiments of ischemia-reperfusion injury and acute rejection.

### 2.3: Surgical Techniques

All surgical procedures were carried out under an operating microscope with 10× to 40× magnification (Leica, Germany). The animals were anesthetized by intraperitoneal injection of 50 mg/kg sodium pentobarbital (Sigma-Aldrich Chemical, USA) and intubated via a tracheotomy with a 22-gauge angiocatheter. The mice were connected to a ventilator (TOPO Small Animal Ventilator, Kent, USA) for respiratory support, using room air at a tidal volume of 0.5-1.0 mL and at a respiratory rate of 120-130/min.

### 2.4: Description of Traditional Procedure

The traditional procedure reported previously by Jungraithmayr [5,10] was used in group 1. Briefly, a median laparosternotomy was performed and the diaphragm was cut along the ventral costal attachment. Next, heparin (100 U/kg) was injected into the inferior vena cava. The donor lung was flushed with 2 mL of cooled (4° C) low-potassium dextran glucose solution from the pulmonary trunk, using a pressure of 10-15 cmH_2_O. The heart-lung block was then harvested at end-tide volume, and the hilum of the left lung was isolated optimally for placement of a cuff with a tail. A hypothermic condition was maintained during placement of a 24-gauge cuff (0.5 mm cuff length with an extending handle of 0.25 mm) into the pulmonary artery and a 22-gauge cuff (0.7 mm cuff length with an extension of 0.3 mm) into the pulmonary vein. A 20-gauge bronchial cuff (1.0 mm cuff length with an extension of 0.5 mm) was not placed until the time of implantation. After exposing and dissecting the recipient’s hilar structures, the pulmonary vessel was clamped by a microvessel clip and the bronchus was unclamped. An incision was made in each of these structures in preparation for the introduction of the cuffs; the low branch of the vein was incised transversely. Anastomosis was completed by grasping the cuff tail, inserting it into the equivalent recipient structure and securing the ligature (10-0 nylon Ethicon Inc.) with forceps.

### 2.5: Description of Improvements

Thirty orthotopic LTs in group 2 were performed with six modifications to the traditional technique.

**Figure 1:**
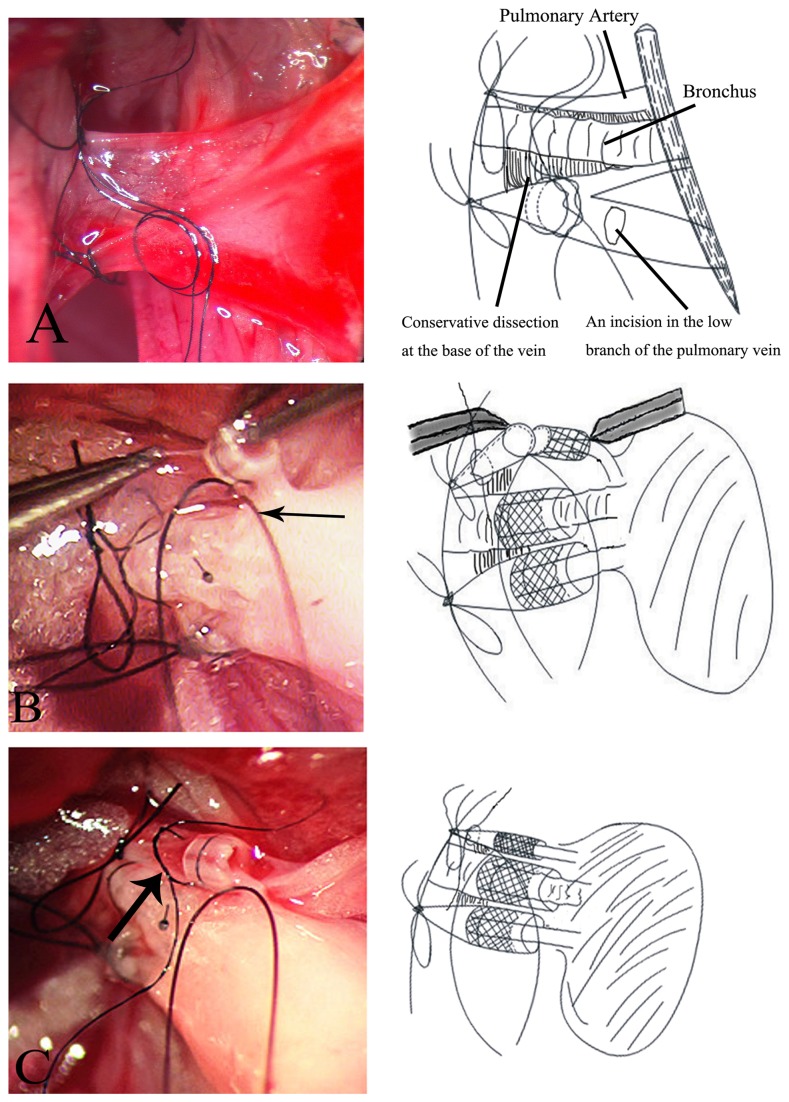
The technique of pulmonary vein anastomosis and the innovation of the cuffed artery insertion. A: After making the conservative dissections at the base of the vessel between the three structures, double circumferential ligatures (10-0 nylon) with the first knot (two rounds) were placed through the tunnel between the vein and anastomotic bronchus to ensure the success of venous anastomosis. B: One side of the incised arterial wall edge was sutured (11-0 silk) and the silk (thin black arrow) was gently fixed to the caudal side. The other side of the wall edge may then be grasped with the blunt tip of forceps and the lumens can be exposed sufficiently to allow easy insertion. C: The adhesive force of the recipient artery kept the cuffed artery from sliding. This simplified the completion of the circumferential ligatures (thick black arrow).

1 The cuff without the tail thoroughly.
2 Shortening the length of the cuff for the vessel and broadening the inner diameter of the cuff for the bronchus. The cuff length for the vessel was 0.5 mm. An 18-gauge angiocatheter, 0.7 mm in length, was used for the bronchus.
3 Dissecting the recipient hilum thoroughly was unnecessary. Small gaps at the base of the vessel (Figure 1, A) were made to place the 8-0 silk (Ethicon Inc.) and preset ligatures.
4 After clamping one side of the circumferential ligature by a microclip, the operator could drag the other side to complete the knot with one hand. Double circumferential ligatures with a first knot (two rounds) were used to ensure successful venous anastomosis (Figure 1, A).
5 One side of the incised arterial wall edge was sutured (11-0 silk Ethicon Inc.), and the silk was gently dragged to the caudal side by a microclip. By holding the other side of the wall edge with forceps and exposing the lumens sufficiently, artery insertion was simplified (Figure 1, B and C).
6 A 24-gauge catheter was inserted into the thorax through the fifth intercostal space before thoracic closure.

### 2.6: Evaluation of Techniques

#### 2.6.1: The Operative Time

The relevant time of the procedure which included warm and cold ischemic times, preparation of cuffs, donor operation and recipient operation was compared between the groups.

#### 2.6.2: Thoracic X-Ray Examination

Chest x-rays were taken on day 30 post-transplant to document changes in the thoracic cavity.

#### 2.6.3: Arterial Oxygen Tension (PaO_2_)

At the time of sacrifice, the animals were anesthetized with pentobarbital sodium, 30 mg/kg, via intraperitoneal injection, then intubated and mechanically ventilated. The thoracic cavity was opened through a median sternotomy and the right lung hilum was clamped for 5 min. 100 μL of blood was collected from the left inferior pulmonary vein via a heparinized syringe and immediately analyzed on a blood gas machine (i-SATA 1-300, Abbott, USA).

#### 2.6.4: Hematoxylin and Eosin (H&E) Staining

The left lungs and normal right lungs from the group 2 were harvested after being flushed with normal saline, and then were post-fixed in 4% paraformaldehyde overnight. The specimens were embedded in paraffin, and glass slides were prepared and heat-fixed. H&E staining was performed.

### 2.7: Statistical Analysis

All data were expressed as mean±SD. The results were analyzed by SPSS 19.0 software. An unpaired t test was used for statistical analysis; a P value of less than 0.05 was considered statistically significant.

## Results

Thirty LTs were performed in group 1, with an 86.7% (26/30) survival rate. One recipient died intraoperatively due to bleeding of the vein during dissection of the hilum; one animal died due to venous thrombosis on day 3 post-operation; and two died due to postoperative pneumothorax and atelectasis.

The survival rate in group 2 was 96.7% (29/30). One death occurred because of a tear in the incised vein during the course of insertion.

### 3.1: The Operative Time

The operative time in group 2 was shorter than that observed using the traditional procedure: 96.75±6.16 min vs. 85.32±6.98 min in groups 1 and 2, respectively (P<0.001, Table 1).

**Table 1:** Comparison of relevant time periods between two groups in the mouse model of orthotopic single lung transplantation (means±SD).

**Procedure**	**Traditional procedure**	**Modified procedure**
	**(MIN±SD, n=10)**	**(MIN±SD, n=15)**
Preparation of cuffs	10.95±1.83	2.23±0.26*
Donor operation	16.85±2.79	16.93±2.76
Recipient operation	56.7±2.59	42.27±5.16*
Total time	96.75±6.16	85.32±6.98*
Cold ischemic time	37.4±1.76	36.07±2.09
Warm ischemic time	33.25±2.61	25.7±5.48*

The improvement techniques saved the time of preparation of cuffs, recipient operation, warm ischemia and total procedure (*P<0.001).

SD, Standard deviation

### 3.2: Macroscopic Appearance of Grafts at the Time of Sacrifice

**Figure 2:**
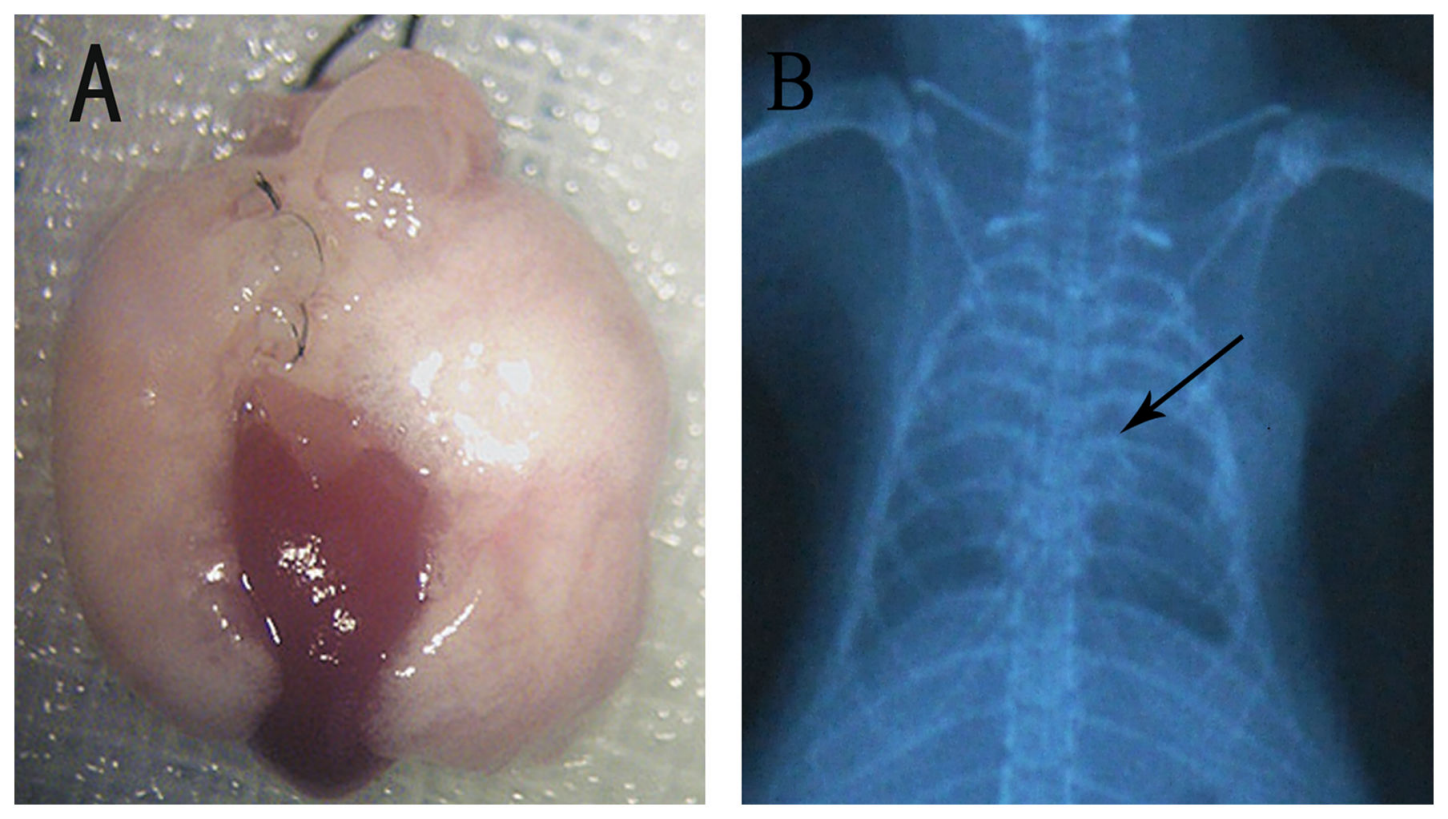
Macroscopic changes (A) and chest radiographs (B) of transplanted recipients in modified group (group 2) at the time of sacrifice after surgery: (black arrow in chest radiographs indicates the cuff). The macroscopic changes of recipients shown perfect perfusion with no lung injury and the chest x-ray examination illustrated that grafts were well inflated on day 30 post-transplant.

On day 30 post-transplant, the grafts showed no lung injury and had perfect perfusion (Figure 2, A).

### 3.3: Thoracic X-Rays

Chest radiography showed that grafts were well inflated on day 30 following surgery (Figure 2, B).

### 3.4: PaO_2_

On day 30 post-transplant, there was no difference of PaO_2_ shown in two groups: 115.9±7.11 mm Hg in group 1 and 116.3±6.87 mm Hg in groups 2 (P>0.05, n=5/group).

### 3.5: H&E Staining

**Figure 3:**
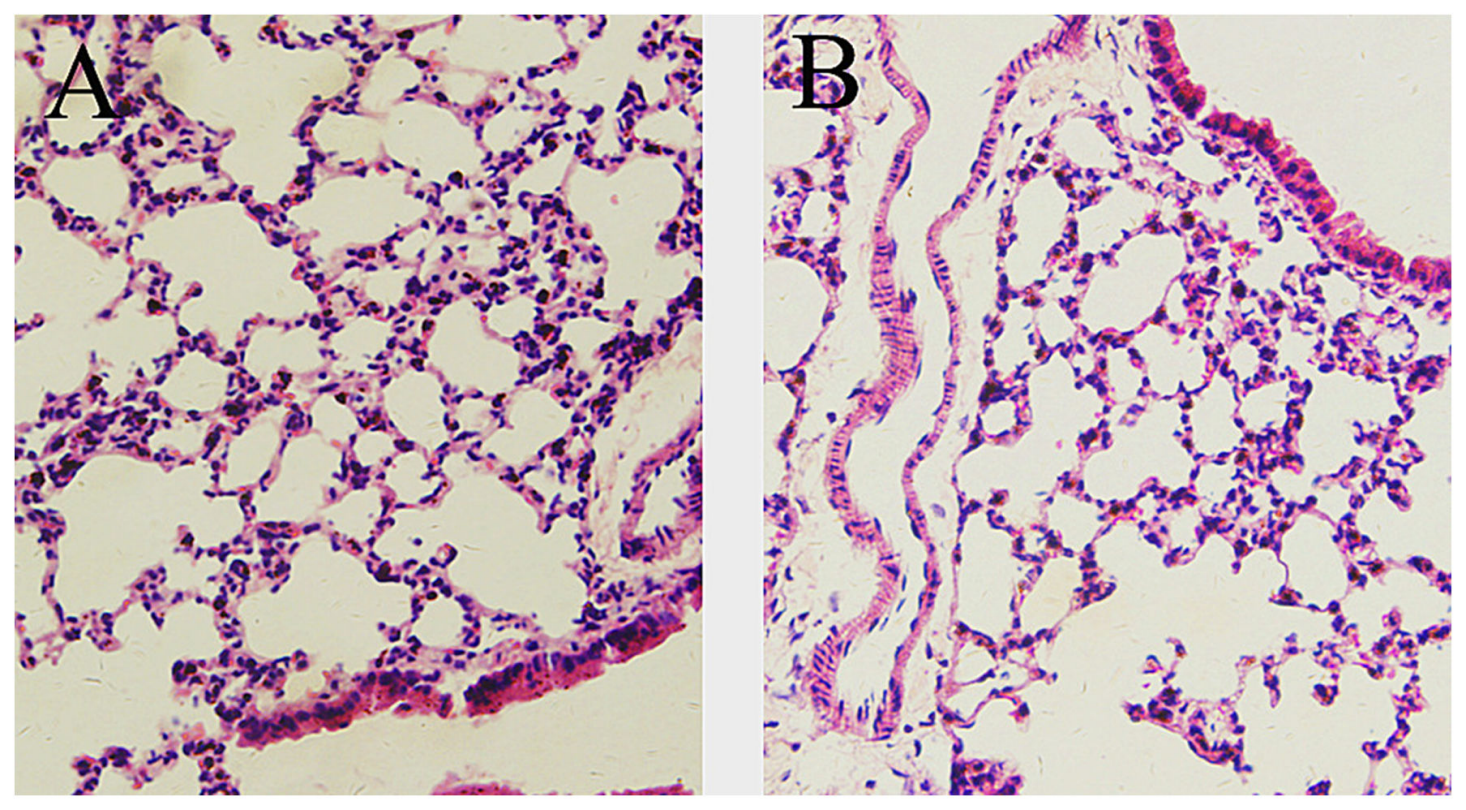
Histological observations in group 2. One month after transplant, lung grafts (A) were histologically similar to that of normal right lungs (B). (400×).

Histologically, the recipients had no evidence of lung injury, pulmonary interstitial lesions, infiltration of neutrophilic granulocytes, or foamy transudates in the alveolar spaces on postoperative day 30 (Figure 3, A), which were similar to that of normal right lungs (Figure 3, B).

## Discussion

Refinements to the traditional mouse orthotopic LT model are needed for the characters of the mouse. In this study, we adopted the modified single-lung transplant technique in the mouse model. In particular, innovative technologies were applied in the securing of the cuffed pulmonary vein and the implantation of the cuffed pulmonary artery, which were not described previously. The results in group 2 in our study demonstrated that the modifications yielded shorter operative times, higher survival rate and fewer complications compared to group 1.

Several potential problems with the cuff-tail technique in the rat model have been reported [7,12]. In that study, the investigators used the cuff tails and cut them off after the anastomosis. In our study, we decided to remove the cuff tail thoroughly for the following five reasons. First, the body of cuff can replace the tail for holding and insertion satisfactorily. Meanwhile, it is difficult and time consuming to remove the handle of the cuff after the procedure is completed. Second, the tail compresses the blood vessels, especially the pulmonary vein. Excessive length of the cuff may lead to lung ischemia or congestion because the vessels in mice are much shorter (one tenth the length) than that in rats. Third, the cuff tails limit the donor implantation direction during anastomosis. Thus, cuff tails may cause reverse anastomosis and result in surgical failure. However, for cuffs without tails, the surgeon can hold any part of the cuff and accomplish anastomosis in accordance with the normal direction of the blood vessel or the bronchus. Fourth, granulomas can form in cuffs, which will result in lumen blockage and dysfunction of the transplanted lung. When the tail is removed, however, fewer foreign-body reactions and less fibrous tissue formation can be expected following transplant surgery. Fifth and last, because of making cuff tails in mice is a more complicated technique, using cuffs without tails simplifies the procedures and decreases the operative time. However, with no tail and shortened cuffs, the venous thrombosis was not observed in group 2.

Hemorrhage is the leading cause of death during the operation. In most cases, it happened in the course of isolating the pulmonary vein. Besides the gentle and careful operative techniques, we made the conservative dissections at the base of the vessel which were enough for placing the 8-0 silk with a slipknot to occlude the left lung blood flow and putting in preset ligatures between the three structures. This modified technique followed the example of method described by our group in the rat model recently [8]. The improvement not only had fewer injuries for the pulmonary vein and the bronchial blood supply, but also reduced operative time compared to that seen with the traditional procedure.

Once the cuff was inserted into the vein, the cuff body, suture, and recipient vein wall should be held in place by a forceps because the lumen of the recipient pulmonary vein lacked the adhesive force. It was difficult to complete the anastomosis by one hand. As a further improvement, we utilized a microclip to clamp one side of the circumferential ligature and dragged it to the other side to complete the knot of the ligature by a forceps simply. In order to prevent the failure of anastomosis, we used double circumferential ligatures with the first knot for a potential salvage procedure.

The diameter of the arterial cuff is much greater than that of the recipient pulmonary artery cavity, so the artery is prone to be torn during the insertion of cuffs, an event that results in surgical failure. To prevent this problem, we sutured (11-0 silk) one side of the incised recipient arterial wall edge and gently pulled the silk to the caudal side by a microclip. The insertion was completed easily by holding the other side of the wall edge with the blunt tip of the forces and exposing the lumen sufficiently. After insertion, the cuff was fixed inside the artery with the help of the tension of the arterial wall. The preset knot could be secured using both hands and the anastomosis of the artery was completed.

As reported by Lin et al. [11], pneumothorax and graft atelectasis were the severe complications in this model. In group 2, to avoid these complications, we inserted a 24-gauge catheter into the thorax through the fifth intercostal space to draw off the residual volume after the pulmonary circulation was restored upon completion of vascular anastomosis. This approach was similar to techniques used in rat orthotopic pulmonary transplantation [12]; on the other hand, we used an 18-gauge cuff instead of the 20-gauge cuff for the bronchus to maintain patency for a longer period of time postoperatively. Assessments of the samples in modified group indicated that all grafts had no atelectasis post-transplant.

In conclusion, the improvements we described above make it easy for researchers to master the procedure technically and perform it confidently. Especially, with the help of innovative technologies for vascular anastomosis, this model could be performed in a successful and reproducible way.
